# Serological Profiling and Neuro-Immune Resilience: The Dissociation Between Anti-SARS-CoV-2 Antibodies and Post-Viral Airway Hyperresponsiveness in Pediatric Asthma

**DOI:** 10.1007/s00408-026-00901-2

**Published:** 2026-06-24

**Authors:** Peter Kunc, Jaroslav Fabry, Jana Mazuchova, Martin Pec, Renata Pecova

**Affiliations:** 1 Department of Pathological Physiology, Jessenius Faculty of Medicine in Martin, Comenius University in Bratislava, Martin, Slovakia; 2 Clinic of Children Tuberculosis and Respiratory Diseases, Jessenius Faculty of Medicine in Martin, Comenius Univesity in Bratislava, Dolny Smokovec, Slovakia; 3 Clinic of Pediatric Respiratory Diseases and Tuberculosis, National Institute of Pediatric Tuberculosis and Respiratory Diseases, Dolny Smokovec, Slovakia; 4 Department of Medical Biology, Jessenius Faculty of Medicine in Martin, Comenius University in Bratislava, Martin, Slovakia

**Keywords:** Pediatric asthma, Long COVID, Cough reflex sensitivity, TRPV1, Inhaled corticosteroids, Neurogenic inflammation

## Abstract

**Background:**

Chronic cough is a frequent symptom of pediatric Long COVID, hypothetically driven by viral neurotropism and sensory nerve sensitization. We investigated the neuro-immune axis in pediatric asthma to determine if the magnitude of post-SARS-CoV-2 humoral immunity correlates with objective airway afferent nerve hypersensitivity.

**Methods:**

This prospective observational study included 61 pre-pubertal children (aged 8 to < 12 years) with well-controlled, predominantly inhaled corticosteroid (ICS)-treated (93.4%) bronchial asthma and confirmed past SARS-CoV-2 infection. Systemic humoral memory was quantified via anti-Spike IgG and IgA titers. Objective cough reflex sensitivity was measured using a capsaicin challenge test, establishing C2 and C5 values. Subjective symptom burden was evaluated using parent-proxy questionnaires (PCQ, VAS, PedsQL).

**Results:**

Stratification by median anti-Spike IgG (125.77 BAU/ml) revealed no significant differences in basal (C2, *p* = 0.301) or motor response (C5, *p* = 0.714) capsaicin thresholds between robust and waning humoral memory states. IgA stratification yielded identical results. Spearman’s correlation confirmed a complete lack of association between absolute IgG titers and neurophysiological markers (*p* > 0.05). Crucially, parent-reported chronic cough severity (PCQ, VAS) and asthma-specific quality of life demonstrated a complete dissociation from objective capsaicin thresholds across all evaluated domains (all *p* > 0.05). Supplementary subgroup analysis revealed no significant differences in cough thresholds based on acute COVID-19 severity (*p* > 0.05).

**Conclusion:**

A robust post-viral humoral immune response to SARS-CoV-2 does not precipitate peripheral airway nerve hypersensitivity in properly controlled, ICS-treated asthmatic children. The complete uncoupling of subjective parent-reported symptoms from objective neurophysiology cautions against diagnosing neurogenic Long COVID based solely on questionnaires, emphasizing the necessity of objective testing and evaluation of alternative atopic etiologies.

## Introduction

The emergence of the post-acute sequelae of COVID-19 (PASC), widely recognized as Long COVID, has presented a persistent challenge in respiratory and immunological medicine. Among the most frequent manifestations of PASC is a chronic, refractory cough, which significantly impairs patients’ quality of life. Emerging clinical and pathophysiological evidence suggests that SARS-CoV-2 exhibits pronounced viral neurotropism, capable of directly or indirectly invading and irritating both the central and peripheral nervous systems [1]. In the respiratory tract, this neuro-inflammatory cascade is hypothesized to alter the sensitivity of airway afferent nerve endings. Specifically, the virus and the subsequent localized immune response can lower the activation threshold of transient receptor potential channels, leading to persistent neurogenic inflammation and cough hypersensitivity syndrome [2].

Children diagnosed with bronchial asthma represent a population theoretically at an elevated risk for such post-viral complications. Pediatric asthma is predominantly characterized by type-2 (T2) inflammation, driven by an intricate interplay of eosinophils, elevated immunoglobulin E (IgE), and specific cytokines [3]. This pre-existing inflammatory milieu chronically primes airway afferent nerves, manifesting clinically as baseline airway hyperresponsiveness. It is well established that common respiratory viruses typically act as potent exacerbators of this asthmatic neurogenic inflammation [4]. Therefore, a superimposed SARS-CoV-2 infection theoretically possesses the capacity to further amplify this neural sensitization.

However, the pediatric immunological response to SARS-CoV-2 diverges significantly from that observed in adult cohorts. Children often mount a highly robust and distinct innate and adaptive immune response, which typically results in milder acute disease but leaves questions regarding long-term neuro-immune interactions [5]. While systemic humoral responses specifically the generation of anti-Spike immunoglobulin G (IgG) and immunoglobulin A (IgA) serve as reliable biomarkers of past viral encounter, disease severity, and immunological memory, their direct correlation with post-viral peripheral neural damage in the pediatric respiratory tract remains poorly elucidated [6]. Furthermore, the majority of current pediatric PASC research relies heavily on subjective symptom questionnaires, which frequently fail to align with objective biological markers, particularly in cases involving chronic cough [7].

To address this critical gap in knowledge, this study aimed to investigate the neuro-immune axis in pediatric asthma by objectively quantifying cough reflex sensitivity (CRS) and correlating it with comprehensive serological profiling. Specifically, we sought to determine whether the magnitude of post-viral humoral response evidenced by high serum titers of anti-Spike IgG and IgA translates into objective peripheral airway nerve sensitization (measured via capsaicin challenge) compared to asthmatic children with lower humoral memory. Additionally, we evaluated whether the underlying atopic burden (total IgE and absolute eosinophil count) influences this post-viral neurogenic trajectory.

## Materials and Methods

### Study Design and Ethical Considerations

This prospective, monocentric, observational study was conducted between March 2024 and February 2026 at the Clinic of Pediatric Respiratory Diseases and Tuberculosis, National Institute of Pediatric Tuberculosis and Respiratory Diseases in Dolny Smokovec, Slovakia, in collaboration with the Jessenius Faculty of Medicine in Martin, Comenius University in Bratislava. The study protocol received dual ethical approval: it was approved by the Ethics Committee of the Jessenius Faculty of Medicine (Approval No. EK 17/2022) and the institutional Ethics Committee of the National Institute (Approval ID: 0212023). The research was conducted in strict accordance with the Declaration of Helsinki and its later amendments. Written informed consent was obtained from the legal guardians of all participating children, accompanied by the verbal or written assent of the children, commensurate with their age and comprehension level.

### Study Population and Baseline Characteristics

The study cohort comprised 61 children (aged 8 to < 12 years, including exclusively pre-menarcheal girls) with an established diagnosis of mild-to-moderate persistent bronchial asthma. This specific age window was selected to ensure adequate cognitive and motor cooperation during complex functional assessments while avoiding the confounding hormonal influences of puberty. To ensure cohort homogeneity and minimize acute inflammatory confounders, strict inclusion criteria were applied. Eligible participants were required to have a confirmed diagnosis of bronchial asthma (predominantly T2 high or T2 low endotype; ICD-10 codes J45.0, J45.9) established by a specialist according to the 2025 Global Initiative for Asthma (GINA) guidelines [8]. The disease had to be controlled or partially controlled, objectively defined by an Asthma Control Test (ACT) score of > 19, despite regular maintenance therapy with inhaled corticosteroids (ICS), ICS/LABA combinations, leukotriene receptor antagonists, and/or antihistamines (GINA treatment step 2–3). To eliminate the influence of acute exacerbations, a wash-out period of at least 30 days without a severe asthma exacerbation and 14 days without an acute respiratory tract infection was mandated prior to functional testing. Children with severe uncontrolled asthma, congenital respiratory anomalies, chronic non-asthmatic pulmonary diseases (e.g., cystic fibrosis, primary ciliary dyskinesia), severe gastroesophageal reflux disease (GERD) requiring surgical intervention, or an inability to properly cooperate with functional maneuvers were excluded from the study.

To rigorously exclude any confounding acute respiratory infections (e.g., RSV, rhinovirus) at the time of functional assessment, every patient underwent a dual clinical evaluation by a pediatric pulmonologist (at enrolment and immediately prior to testing) confirming the absence of acute upper or lower respiratory tract symptoms. Furthermore, acute subclinical inflammation was excluded via standard laboratory screening (normal complete blood count with differential, C-reactive protein < 10 mg/L, and normal erythrocyte sedimentation rate). Routine multiplex PCR respiratory viral panels were not performed, reflecting standard real-world outpatient practice for asymptomatic, stable children. Regarding upper airway comorbidities, among the 50 patients (82.0%) diagnosed with mild persistent allergic rhinitis, none received allergen immunotherapy or topical nasal antihistamines. All 50 patients were prescribed intranasal corticosteroids (INCS) strictly on an ‘on-demand’ basis, and a mandatory washout period of at least 14 days without INCS use was required prior to capsaicin testing.

The baseline demographic, clinical, and pharmacological characteristics of the enrolled study population are summarized in Table 1. The mean age of the participants was 9.7 ± 1.5 years, with a gender distribution of 42 boys (68.9%) and 19 girls (31.1%). The mean body mass index (BMI) was 18.7 ± 4.4 kg/m^2^. Notably, based on age- and gender-specific pediatric growth charts, none of the enrolled patients met the criteria for obesity (defined as BMI > 97th percentile), effectively eliminating obesity-induced systemic meta-inflammation and altered respiratory mechanics as potential confounders for cough reflex hypersensitivity. Detailed phenotyping of the cohort revealed a high prevalence of type-2 inflammatory features, consistent with the asthmatic terrain. The overall atopic status was positive in 50 patients (82.0%), with allergic rhinitis being the most frequent comorbidity (50 patients, 82.0%). GERD, a known non-asthmatic trigger for neurogenic cough, was present in 41 patients (67.2%).

**Table 1:** Baseline demographic, clinical, and pharmacological characteristics of the study population (N = 61)

Characteristic	Value
Demographics	
Age (years), mean ± SD	9.7 ± 1.5
Gender, n (%)	
Boys	42 (68.9%)
Girls	19 (31.1%)
BMI (kg/m^2^), mean ± SD	18.7 ± 4.4
Clinical phenotype & comorbidities	
Bronchial asthma, n (%)	61 (100.0%)
Atopic status positive, n (%)	50 (82.0%)
Allergic rhinitis, n (%)	50 (82.0%)
GERD, n (%)	41 (67.2%)
Maintenance Therapy (GINA step 2–3)	
Inhaled corticosteroids (ICS), n (%)	57 (93.4%)
Long-acting β2-agonists (LABA), n (%)	22 (36.1%)
Antihistamines, n (%)	53 (86.9%)
Montelukast (LTRA), n (%)	26 (42.6%)

Data are presented as mean ± standard deviation (SD) for continuous variables and as absolute numbers (n) with percentages (%) for categorical variables. BMI, Body Mass Index; GERD, Gastroesophageal Reflux Disease; ICS, Inhaled Corticosteroids; LABA, Long-Acting Beta-Agonists; LTRA, Leukotriene Receptor Antagonists

Crucially for the subsequent interpretation of the outcomes, the cohort was highly homogenous regarding anti-asthmatic maintenance therapy. A vast majority of the patients (57, 93.4%) were treated with ICS, either as monotherapy or in combination with long-acting β2-agonists (LABA; 22, 36.1%). Additional pharmacological management included oral antihistamines (53, 86.9%) and leukotriene receptor antagonists (26, 42.6%). This profound homogeneity in ICS-based controller therapy ensures that the baseline anti-inflammatory suppression of the airway afferent nerve terminals was comparable across the cohort, thereby isolating the potential neurotropic effect of the SARS-CoV-2 infection.

Regarding the SARS-CoV-2 infection history, all children had a confirmed previous infection. As detailed in Table 2, the acute phase of the disease was predominantly mild (20 patients, 32.8%) or asymptomatic (4 patients, 6.6%), with only a minority experiencing moderate (13 patients, 21.3%) or severe (3 patients, 4.9%) courses. For the remaining 21 patients (34.4%), the exact severity of the acute phase was not specified in the available medical records. Parent-reported persistent Long COVID symptoms (lasting > 4 weeks) were noted in 7 patients (11.5%). Furthermore, 2 patients (3.3%) had received at least one dose of a COVID-19 vaccine prior to the assessment.

**Table 2:** SARS-CoV-2 infection history and Long COVID status of the study population

Characteristic	Value
COVID-19 History	
SARS-CoV-2 infection confirmed, n (%)	61 (100.0%)
Acute disease severity, n (%)	
Asymptomatic	4 (6.6%)
Mild	20 (32.8%)
Moderate	13 (21.3%)
Severe	3 (4.9%)
Unknown / Not reported	21 (34.4%)
Parent-reported Long COVID (> 4 weeks), n (%)	7 (11.5%)
COVID-19 vaccination (≥ 1 dose), n (%)	2 (3.3%)

### Serological and Immunological Profiling

To objectively categorize the temporal proximity and intensity of the post-viral immune response, comprehensive serological testing was performed from venous blood samples collected under standardized fasting conditions. The serum levels of specific anti-SARS-CoV-2 Spike receptor-binding domain (RBD) antibodies immunoglobulin G (IgG) and immunoglobulin A (IgA) were quantified using commercially available enzyme-linked immunosorbent assays (CORG96 EIA COVID-19 RBD IgG and CORA96 EIA COVID-19 RBD IgA; TestLine Clinical Diagnostics, Brno, Czech Republic). The results were expressed in Binding Antibody Units per milliliter (BAU/ml). To assess the underlying atopic and type-2 (T2) inflammatory milieu, absolute peripheral blood eosinophil counts (× 10^9/L) and total serum IgE levels (IU/ml) were measured using standard automated laboratory analyzers.

### Cough Reflex Sensitivity Testing

Objective quantification of airway afferent nerve sensitivity was conducted utilizing a standardized capsaicin challenge test, adhering to established European Respiratory Society guidelines and our previously described institutional protocol [9]. The provocation agent, capsaicin (Sigma-Aldrich, St. Louis, MO, USA), was dissolved in physiological saline with 10% ethanol and subsequently diluted to create a series of 12 doubling concentrations ranging from 0.61 to 1250 µmol/L. Following a baseline inhalation of physiological saline, capsaicin aerosols were administered via a dosimeter (KoKo DigiDoser; nSpire Health, Longmont, CO, USA). Subjects inhaled the aerosol via a mouthpiece using a single, deep inspiratory vital capacity maneuver. The number of coughs elicited immediately after each inhalation was manually counted by an experienced physician. The primary endpoints defining CRS were established as the lowest provocative capsaicin concentration capable of inducing at least two (C2 value) and at least five (C5 value) coughs. Because capsaicin thresholds exhibit a log-normal distribution, the threshold values were logarithmically transformed (log_10_) for statistical analyses.

### Pulmonary Function and Clinical Assessments

Baseline airway obstruction and local eosinophilic airway inflammation were evaluated prior to the CRS testing to avoid provocation-induced artifacts. Fractional exhaled nitric oxide (FeNO) levels were measured using a portable NIOX VERO® device (Aerocrine AB, Solna, Sweden). according to standard ATS [10]. Spirometric evaluations (FEV1 and FVC) were performed using a calibrated spirometer and expressed as percentages of predicted normal values based on the Global Lung Function Initiative (GLI) reference equations [11].

To contrast objective physiological measurements with subjective patient- and parent-reported outcomes, three validated instruments were utilized. The Pediatric Cough Questionnaire (PCQ), a parent-proxy tool originally developed by Paul et al., assessed nocturnal cough frequency, sleep disruption, severity, and overall bothersomeness on a 0–30 scale [12]. Asthma-specific health-related quality of life was evaluated using the parent-proxy report of the PedsQL™ 3.0 Asthma Module, transformed to a 0–100 scale [13]. Additionally, the overall subjective intensity of chronic cough was measured via a 10-point Visual Analogue Scale (VAS). Finally, a detailed clinical history was obtained to identify parent-reported, persistent “Long COVID” symptoms exceeding four weeks post-infection.

### Statistical Analysis

Data management and statistical analyses were performed using standard software (The Jamovi Project, 2023, Sydney, Australia, version 2.3.28.0). The normality of data distribution was assessed using the Shapiro–Wilk test. Due to the predominantly non-normal distribution of the clinical and immunological variables, particularly the capsaicin thresholds, non-parametric methods were applied. The study cohort was stratified into two comparative groups based on the median values of anti-Spike IgG and IgA, representing a “robust/high” versus a “waning/low” immunological memory state. Group comparisons were conducted using the Mann–Whitney U test for continuous variables and Fisher’s exact test for categorical variables. Correlations between serological markers, atopic burden, and CRS were evaluated using Spearman’s rank correlation coefficient. A *p* value of < 0.05 was considered statistically significant.

A post-hoc power analysis was conducted to validate the robustness of null findings. With a sample size of N = 61, the study provided greater than 80% power (α = 0.05) to detect a medium effect size (Cohen’s d = 0.5), corresponding to a clinically relevant difference of one capsaicin doubling dose between the groups.

## Results

### Objective Cough Reflex Sensitivity and Serological Status

To evaluate the direct physiological impact of the SARS-CoV-2 infection on airway afferent nerve excitability, the study cohort (*N* = 61) was stratified based on the median serum anti-Spike IgG level (125.77 BAU/ml). This generated a High IgG group (*n* = 31), indicative of a more robust humoral response to infection, and a Low IgG group (*n* = 30), indicative lower humoral memory.

Objective quantification of airway afferent nerve sensitivity via the capsaicin challenge demonstrated no statistically significant differences between the groups. The geometric mean (GMean) of the C2 value representing the basal excitability of the cough reflex was 8.50 µmol/L in the High IgG group compared to 11.90 µmol/L in the Low IgG group. Despite a numerical trend towards lower values (higher sensitivity) in the High IgG group, this difference did not reach statistical significance (*p* = 0.301). Furthermore, the C5 value, which represents the motor response to more intense nociceptive stimulation, was remarkably comparable between the two groups (High IgG GMean: 35.72 µmol/L vs. Low IgG GMean: 40.91 µmol/L, p = 0.714).

**Fig. 1:**
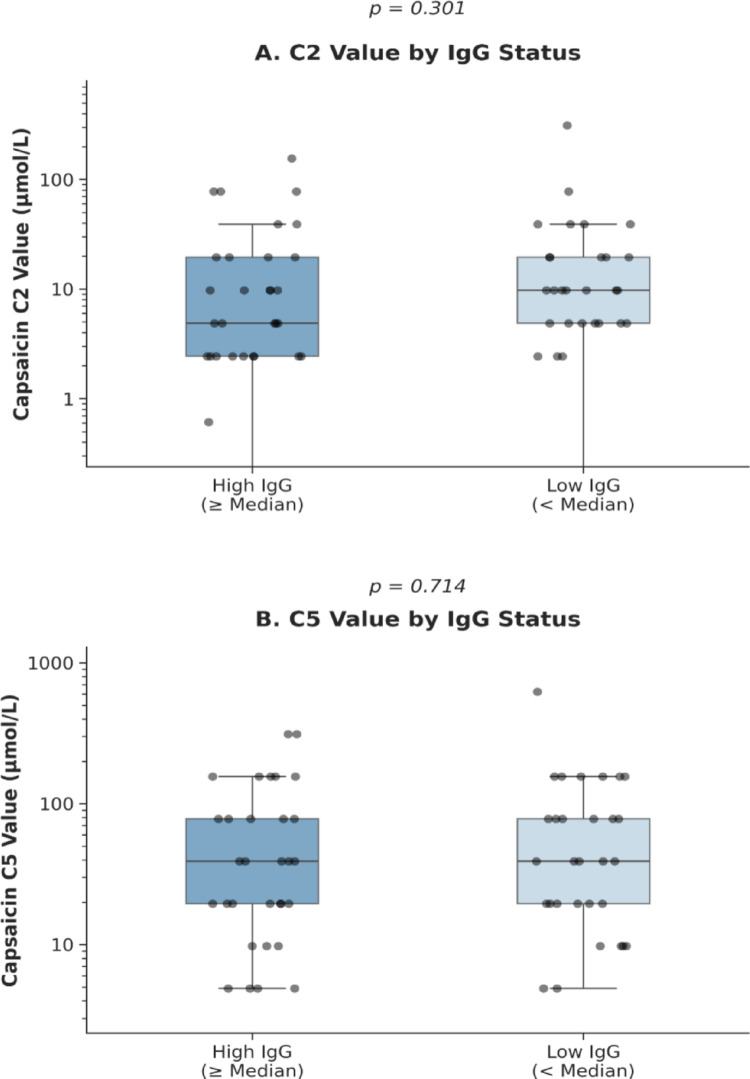
Objective cough reflex sensitivity grouped by post-viral systemic humoral memory. Capsaicin C2 (**A**) and C5 (**B**) values are stratified by median anti-SARS-CoV-2 IgG levels. Boxplots display medians and interquartile ranges on a logarithmic scale, overlaid with individual patient scatter plots

**Fig. 2:**
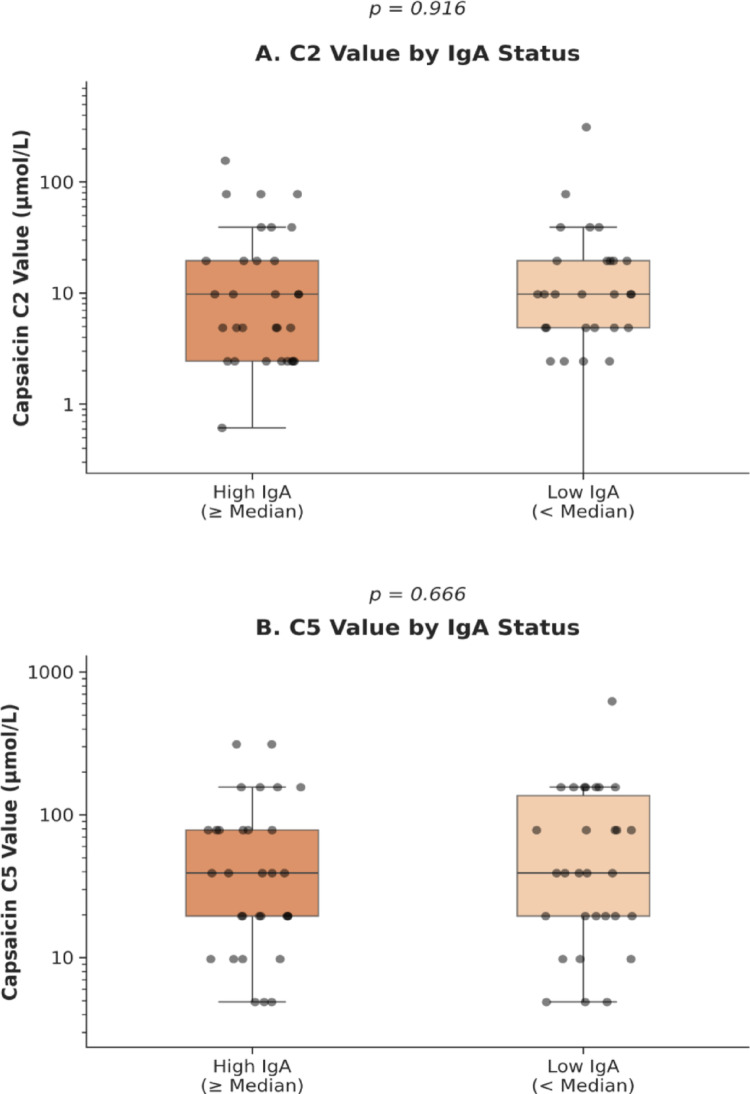
Objective cough reflex sensitivity grouped by post-viral mucosal immune response. Sensitivity analysis depicting capsaicin C2 (**A**) and C5 (**B**) values stratified by median anti-SARS-CoV-2 IgA levels

To ensure the robustness of these findings and to account for the specific mucosal immune response, a parallel sensitivity analysis was performed using serum anti-Spike IgA stratification (median cut-off: 59.57 BAU/ml; High IgA *n* = 31, Low IgA *n* = 30). The IgA-based analysis entirely corroborated the IgG findings, demonstrating no statistically significant differences in either the C2 (p = 0.916) or C5 (p = 0.666) capsaicin values between the cohorts. The detailed visualization of these relationships is presented in Fig. 1 (systemic IgG response) and Fig. 2 (mucosal IgA response).

A supplementary subgroup analysis evaluating the impact of acute COVID-19 severity was performed on 40 patients with fully documented acute phase records. Comparing patients with mild/asymptomatic acute courses (n = 24) against those with moderate/severe acute courses (n = 16) revealed no significant differences in either C2 (p = 0.736) or C5 (p = 0.416) capsaicin thresholds.

### Subjective Post-Viral Symptom Burden and Quality of Life

We subsequently analyzed whether the divergent systemic serological status translated into alterations in subjective, parent-reported clinical outcomes. Assessment of nocturnal cough frequency, sleep disruption, and overall bothersomeness utilizing the Pediatric Cough Questionnaire (PCQ) yielded a mean score of 5.10 in the High IgG group versus 2.73 in the Low IgG group. While the High IgG group exhibited slightly higher mean symptom scores, this difference was not statistically significant (p = 0.149). Similarly, the overall subjective evaluation of chronic cough via the Visual Analogue Scale (VAS) demonstrated no significant divergence between the groups (High IgG: 2.53 vs. Low IgG: 3.40, p = 0.420).

Crucially, the asthma-specific health-related quality of life, measured via the PedsQL™ Asthma Module (where higher scores indicate better clinical control and fewer symptoms), remained well-preserved and statistically indistinguishable across the immunological strata (High IgG: 69.18 vs. Low IgG: 66.77, p = 0.654).

### Correlation Analysis Between Humoral Immunity and CRS

To evaluate the neuro-immune axis as a continuous spectrum and rule out any potential non-linear dependencies not captured by median stratification, Spearman’s rank-order correlation analysis was conducted between the absolute titers of anti-SARS-CoV-2 antibodies and objective capsaicin values.

The analysis revealed a complete lack of a significant linear or monotonic relationship. Specifically, circulating anti-Spike IgG levels exhibited no significant correlation with either the C2 value (*ρ* = − 0.076, *p* = 0.566) or the C5 value (*ρ* = − 0.046, *p* = 0.726). These correlational findings definitively support the group-based comparisons, confirming that the magnitude of the post-viral humoral immune response is not a predictor of peripheral airway nerve sensitization in properly controlled asthmatic children.

### Correlation Analysis of Neurophysiological, Immunological, and Clinical Parameters

To evaluate the neuro-immune axis as a continuous spectrum and rule out any potential non-linear dependencies not captured by median stratification, Spearman’s rank-order correlation analyses were conducted. This approach simultaneously assessed the alignment between objective neurophysiology (capsaicin C2 and C5 values), the magnitude of the humoral immune response, and parent-reported symptom severity.

The analyses, detailed in Table 3, demonstrated a complete uncoupling across all evaluated domains. Specifically, absolute titers of circulating anti-Spike IgG exhibited no significant linear or monotonic relationship with either the basal or motor response cough reflex thresholds. Furthermore, parent-reported symptom severity failed to reflect the underlying airway afferent nerve sensitivity. There was no significant correlation between the subjective perception of cough (PCQ total score, VAS intensity) and objective peripheral nerve sensitization. Similarly, the asthma-specific and total health-related quality of life (PedsQL) showed no dependency on the physiological excitability of the cough reflex.

**Table 3:** Spearman’s rank correlation analysis between objective cough reflex sensitivity (capsaicin C2 and C5 values) and both systemic humoral immunity and subjective clinical outcomes

Parameter	Objective CRS threshold	Spearman’s ρ	*p* value	n
immunological markers				
Anti-spike IgG (BAU/ml)	C2 (log µmol/L)	− 0.076	0.566	61
Anti-spike IgG (BAU/ml)	C5 (log µmol/L)	− 0.046	0.726	61
Subjective clinical metrics				
PCQ total score	C2 (log µmol/L)	− 0.047	0.728	58
PCQ total score	C5 (log µmol/L)	0.157	0.228	61
VAS cough intensity	C2 (log µmol/L)	− 0.027	0.843	57
VAS cough intensity	C5 (log µmol/L)	0.061	0.642	60
PedsQL (asthma module)	C2 (log µmol/L)	0.048	0.722	58
PedsQL (asthma module)	C5 (log µmol/L)	-0.108	0.407	61
PedsQL (total score)	C2 (log µmol/L)	0.082	0.540	58
PedsQL (total score)	C5 (log µmol/L)	-0.141	0.280	61

## Discussion

The primary objective of this study was to objectify the neuro-immune axis in pediatric asthma following SARS-CoV-2 infection by evaluating the relationship between post-viral humoral immunity and airway afferent nerve excitability. The principal finding demonstrates a profound neuro-immune resilience in this cohort: neither the magnitude of the predominantly systemic (IgG) nor predominantly mucosal (IgA) anti-Spike antibody response correlated with objective peripheral airway nerve sensitization. These findings align with recent large-scale pediatric cohorts demonstrating that robust and durable anti-Spike IgG responses in children reflect adaptive immune memory, rather than serving as predictive biomarkers for the presence or severity of Long COVID sequelae [14, 15].

Crucially, capsaicin C2 and C5 values provide a highly specific, direct functional readout of transient receptor potential vanilloid 1 (TRPV1) channel excitability on vagal C-fibers, as robustly demonstrated by prior clinical trials utilizing targeted TRPV1 antagonists [16, 17]. Current neuro-immunological literature establishes that the adaptive immune response and peripheral neuroplasticity are mechanistically linked; viral challenges and subsequent cytokine networks (e.g., IL-6, interferons) can bridge the blood–brain barrier and drive profound neuronal hyperexcitability and synaptic remodeling [18–20]. However, the absolute lack of correlation between systemic antibody titers and objective capsaicin thresholds in our study introduces a vital clinical nuance. It demonstrates that while these systems undoubtedly interact during the acute inflammatory phase, a robust and persistent humoral footprint does not obligatorily signal ongoing peripheral neuroplasticity [21–23]. Consequently, although systemic antibodies reliably quantify the B-cell response to past infection, their elevated titers do not imply a persistent neurogenic scar. They are, therefore, invalid as surrogate biomarkers for post-viral cough hypersensitivity.

The absence of capsaicin-induced hypersensitivity in children with strong humoral memory must also be critically interpreted within the context of their baseline pharmacological management. In our cohort, 93.4% of patients were receiving regular inhaled corticosteroids (ICS) as standard controller therapy. While there is no direct evidence that ICS specifically downregulates transient receptor potential (TRP) channels, respiratory viruses are known to upregulate TRPV1 and TRPA1 expression on sensory neurons primarily via virus-induced soluble factors and inflammatory cytokines (e.g., IL-6, IL-8) [24]. We hypothesize that pre-existing and continuous ICS therapy acts as a “pharmacological shield.” By profoundly suppressing the initial localized inflammatory cytokine storm in the airway epithelium, ICS likely prevents the secondary, cytokine-driven neuroplastic upregulation of TRPV1 channels, thereby preserving the basal excitability of the cough reflex.

In addition to pharmacological suppression, the inherent characteristics of the pediatric innate immune system likely contribute to this observed resilience. Unlike adults, who frequently exhibit prolonged systemic hyperinflammation and delayed viral clearance, pediatric patients possess pre-activated airway epithelial cells and mount a rapid, vigorous mucosal interferon response [25, 26]. This highly efficient initial containment restricts SARS-CoV-2 replication predominantly to the superficial epithelial layers. Consequently, this prevents deep tissue penetration and mitigates the risk of direct cytopathic invasion of subepithelial vagal nerve endings, a mechanism frequently implicated in adult post-viral neuropathies [26].

Perhaps the most clinically consequential finding of our study lies in the complete dissociation between parent-reported symptom scores (PCQ, VAS) and objective neurophysiology (Table 3). While parent-proxy reports suggested persistent cough in a subset of our patients, capsaicin thresholds confirmed the absence of objective airway afferent hypersensitivity. This disconnect directly mirrors recent systematic reviews highlighting that pediatric Long COVID symptom reporting is frequently discordant with objective clinical, imaging, or pulmonary function abnormalities [27, 28]. Given the exceptionally high prevalence of allergic rhinitis (82%) within our cohort, it is highly probable that the subjectively reported “post-COVID cough” is driven mechanically by upper airway cough syndrome (UACS) or pre-existing atopic traits, rather than by a novel post-viral vagal neuropathy. Furthermore, subjective reporting may be heavily influenced by pandemic-related psychosocial stress and caregiver recall bias [27, 29]. This uncoupling serves as a critical warning against the premature attribution of common pediatric respiratory phenotypes to PASC without objective physiological verification.

This study possesses several methodological limitations. The cross-sectional evaluation of immunological memory prevents us from establishing a precise temporal trajectory from acute infection to functional testing. Furthermore, while the capsaicin challenge is the gold standard for assessing cough reflex sensitivity, clinical trials utilizing TRPV1 antagonists have demonstrated that normalized capsaicin thresholds do not always translate to a reduction in spontaneous daily cough frequency, adding nuance to the interpretation of cough dynamics in broader context [16]. Additionally, the lack of routine multiplex PCR viral panels at the time of testing prevents the absolute exclusion of recent subclinical viral encounters, although strict clinical and laboratory gating minimized this risk. The supplementary subgroup analysis regarding COVID-19 severity must also be interpreted with caution, as the relatively small number of moderate/severe cases renders this specific comparison underpowered. Finally, the monocentric design and the specific inclusion of tightly controlled, ICS-treated pre-pubertal asthmatics limit the external validity of our findings. We cannot extrapolate these results to therapy-naïve children or those with severe, uncontrolled asthma phenotypes.

## Conclusion

This study provides objective evidence that an intense post-viral humoral immune response to SARS-CoV-2 does not translate into peripheral airway nerve hypersensitivity in children with well-controlled, ICS-treated bronchial asthma. The objective preservation of the cough reflex, coupled with a complete lack of correlation with parent-reported symptoms, emphasizes a robust neuro-immune resilience in this population. Our findings highlight the profound limitation of relying solely on subjective questionnaires for diagnosing pediatric Long COVID. Clinicians must prioritize objective physiological testing and rule out highly prevalent alternative etiologies, such as allergic rhinitis, before attributing chronic pediatric cough to SARS-CoV-2 induced neuropathy.

## Data Availability

The raw datasets generated for this study are available on request to the corresponding author. The data are not publicly available due to privacy and ethical restrictions.
